# Address before the Michigan Dental Association

**Published:** 1865-02

**Authors:** G. W. Stone


### ADDRESS,

BY THE RETIRING PRESIDENT, G. W. STONE, BEFORE THE

### MICHIGAN DENTAL ASSOCIATION.

*Mr. President and Gentlemen of the Association:*

In accordance with the laws and customs which bind us together in our professional association, it becomes my duty to make a few remarks appropriate to the occasion, as well as to give my feeble testimony upon some one of the many subjects which belong to, and are calculated to interest the different members of the dental profession.

And I am sure, gentleman, that of one thing, at least, I can feelingly speak, and that is, to express to you my heartfelt thanks for the courtesy, the unfailing kindness, the indulgent forbearance which has been extended toward me from each and all of you while striving to perform to the best of my abilities, the various and important duties which pertain to the office of President of this Association. At the same time I am aware that I had no right to expect aught else from a body of men occupying the high position to which the Dental profession has risen in this country. For it appertaineth to gentlemen to be courteous, kind and forbearing of the weaknesses of others. For your patience and forbearance during my administration please accept my thanks.

And while I cheerfully vacate the chair of President, I gladly welcome my successor, hailing him as one worthy of the position—an earnest, skillful and experienced dentist, as well as a high-minded gentleman.

And here, gentlemen, I would gladly pause, but, alas, custom and expectation, both alike, demand that I should proceed to express my views upon some one of the many appropriate subjects which I may choose and select.

I would therefore invite your attention to a few remarks upon "Dental Association," and by this term I intend to nclude both the occasional gatherings (like the present) of the profession for the purpose of mutual conference and consultation, and that of visits in leisure hours to the rooms and laboratories of our brethren in the profession.

The advancement of the profession, the advantages of mutual combination and conference; the interchange of our various experience; the exhibition of the results of our study; the discussion of theories; the display of inventions; the opportunities for friendly and professional intercourse; all these were well understood, else why was this association formed. Why are we gathered here to day? Why are you listening to my voice, while I take a brother's privilege to trespass upon your time and attention? The study of dentistry has now become a science, being the chosen avocation of thousands of the skillful and eminent minds of the age, employing many and various trades to supply its needs; having gained for the members of the profession a high and honored position in the confidence of the people. And so it was found necessary to join together in mutual association, not only to promote the ends already mentioned, but also to protect the public and the profession from a host of quacks, who, without any scientific knowledge, hardly knowing a molar from an incisor; having no stock but brass, tin foil, a tooth pick and a turnkey, set up for practical dentists, and by dint of advertising and a large show of wonderful teeth in a tin cup, one of which was said to be filled on a wager with some dentist far away, who, though skillful, failed to win the prize, strove to rob the learned of the profession of the due award of his labor, and the honor due to the study of years and the labor of a lifetime. Therefore, there was need of an association; of a joining together for mutual protection and advancement; for friendly intercourse and scientific investigation. For all these purposes we have associated ourselves together, and long may our association continue to exist to our mutual honor and profit.

I will now pass on to my second head, namely, the advantage and need of a mutual interchange of courtesies and kindnesses between those of the same profession in a private and semi-confidential way as well as in our public conference and consultation.

Of course it is needless for me to say aught about the advantage of making friends of those whose lives and occupations are so nearly like our own. I shall therefore merely present a few thoughts upon the benefit and advantage of professional visits to the laboratories and operating rooms of our brethren in the profession.

It is our lot, in common with that of every other noble profession, to have in our midst a class of men, who, though *with* us are not *of* us; who are mean, penurious and uncharitable; who have no love for their profession, but merely get their living by it; who are envious, suspicious, and regardless of the feelings or interests of others. You visit the room of such a man. There is a patient in the chair. You take a seat in the ante-room while the operation is being completed; or if there is no ante-room, the operation is postponed and the patient is told to come some other time; the instruments are all put away, and you are anxiously asked about the weather, the crops, or the health of your wife and family, He looks upon you as a spy in the goodly field where he gathers his harvest of greenbacks; and you are soon made to feel that your visit is an unwelcome one. You and I have seen such cases as this. We *know* them to be real.

But let me transport you to a better scene—to the office of one of those men who ornament and dignify the profession. You enter his office. You are met with a kindly grasp and with hearty words of welcome. There is a patient in the chair to whom you are introduced and, by permission, invited to witness the operation. You are shown the different instruments, especially those which he has made for particular operations. There is a new style of forceps that is put in your hand and its use explained, and then the new theories and facts of the science are discussed. When you go home, perhaps you have a few new ideas. You may make one or two better instruments. He has helped you in your work, and at no cost to himself; and when he in turn visits your office, you are glad to see him, and explain to him your own progress and the little things which having made yourself, you especially prize as being a little better than anything else that was ever made for that special purpose. You take him through the whole establishment, Being each of you above the little meanness of envy or professional jealousy, your mutual visits do you good. You are better dentists, for you profit by the experience of others as well as your own. You gain in knowledge; you gain in mutual sympathy and even in professional fame. This is but hinting at the subject; it is the shadow of the reality. You can fill it out for yourselves. Then why not be frequent in the interchange of such courtesies—in the formation of such associations. Why not strive in every way to advance the knowledge and to enhance the skill of your brethren in the profession? Why not be always kind, courteous and considerate?

If a miserable quack and impostor enters your door, have nothing to do with him, except at the end of your boot; but if a true dentist enters, treat him as a gentleman, treat him as one, and exact similar treatment for yourselves. Finally, gentlemen, permit me to close with an expression of my my hearty wish for the happiness, honor and success of each and all of you, and for this association, and may it never die until the last cavity has been filled and the last bad tooth extracted from the month of the last man of the human race.

MUSCATINE, IOWA, March 10th, 1865.

TO THE DENTAL FRATERNITY, IN IOWA.

As this number of the "Dental Register of the West" is intended to reach every member of the profession in our State, I beg leave to call your attention once more to the fact that some of you are not yet up to the standard that every good dentist ought to be in associational effort for the advancement of our profession. The Iowa State Dental Society is now a permanent institution; will meet hereafter annually at such time and place as its members may direct. Our next meeting will take place at Dubuque, Iowa, beginning Tuesday, July 18th, 1865, ; this meeting to be held just one week before the meeting of the "National Dental Association," at Chicago; at which meeting we want Iowa well represented. We therefore call upon every dentist in our State to not let *anything hinder* his presence at this meeting at Dubuque.

We hope all will feel especially called upon to prepare essays on subjects of their own choice and present before the Society.

Yours, fraternally, WILLIAM O. KULP, Cor. Sec'y.

I. S. D. S.

